# miR‐132 mediates a metabolic shift in prostate cancer cells by targeting Glut1

**DOI:** 10.1002/2211-5463.12086

**Published:** 2016-06-08

**Authors:** Wei Qu, Shi‐mei Ding, Gang Cao, She‐jiao Wang, Xiang‐hong Zheng, Guo‐hui Li

**Affiliations:** ^1^ Department of Nuclear MedicineThe Second Affiliated Hospital of Medical School of Xi'an Jiaotong UniversityShaanxiChina; ^2^ Department of EndocrinologyThe Second Affiliated Hospital of Medical School of Xi'an Jiaotong UniversityShaanxiChina; ^3^ Department of General SurgeryThe Second Affiliated Hospital of Medical School of Xi'an Jiaotong UniversityShaanxiChina

**Keywords:** Glut1, glycolysis, miR‐132, prostate cancer cell

## Abstract

Prostate cancer is the second leading cause of cancer‐related deaths among men worldwide. Early diagnosis increases survival rates in patients but the survival rate has remained relatively poor over the past years. Increasing evidence shows that altered metabolism is a critical hallmark in prostate cancer. There is a strong need to explore the molecular mechanisms underlying cancer metabolism for prostate cancer therapy. Whether the aberrant expression of microRNA (miRNA) contributes to cancer metabolism is not fully known. In this study, we found that microRNA‐132 (miR‐132) expression is reduced and thus leads to a metabolic switch in prostate cancer cells. miR‐132 performs this role by increasing Glut1 expression, resulting in the enhanced rate of lactate production and glucose uptake. The altered metabolism induced by decreased miR‐132 levels confers the rapid growth of the cancer cells. These data indicate that miR‐132 is involved in regulating the Warburg effect in prostate cancer by inhibiting Glut1 expression.

**Abbreviations**
2‐DG: 2‐deoxyglucose
3′ UTR: 3′‐untranslated regions
Glut1: glucose transporter 1
IDH3a: isocitrate dehydrogenase 3 (NAD+) alpha
qRT‐PCR: quantitative reverse transcription polymerase chain reaction

Prostate cancer affects one in nine men over the age of 65 and represents the most commonly diagnosed cancer in American men 1. Although radiation and surgery are generally effective for the majority of men, the prognosis remains poor in patients with progressive disease 2. Prostate cancer cells, in particular, are exquisitely sensitive to metabolic changes and to cancer genes that alter metabolic homeostasis 3, 4. A distinct set of genes, proteins, and metabolites orchestrate cancer progression from a precursor lesion to localized disease and finally to metastatic cancer 5. Although gene and protein expression profiles have been extensively studied in prostate cancer, little is known about how prostate cancer cells undergo a metabolic switch or reprogramming. Understanding the metabolic shift in prostate cancer is a key step in the development of new and effective therapeutic approaches.

Altered metabolism is a common feature of cancer cells 6. Normally, noncancerous cells rely primarily on oxidative phosphorylation in the mitochondria to generate adenosine triphosphate for cellular physiology; however, even in the presence of sufficient oxygen, rapidly proliferating cancer cells metabolize large amounts of glucose for lactate, ATP production, carbohydrates, and nucleic acids 7. This phenomenon is known as the ‘Warburg effect’. Tumor cells reprogram their metabolic pathway to meet the rapid growth needs including nucleic acids and glycolytic intermediates. As a result, the metabolic shift toward aerobic glycolysis in cancer cells is believed to be due to the increased demand to support the rapid growth of cancer cells 8. There is an increasing amount of evidence showing that reprogrammed cancer metabolism is a potential target in cancer therapy.

microRNA (miRNA) are endogenous noncoding RNA composed of 18–24 nucleotides that regulate gene expression at the post‐transcriptional level in a sequence‐specific manner 9. miRNA negatively suppress gene expression by complementarily binding to the 3′‐untranslated regions (3′UTR) that results in the degradation of target mRNA or the repression of mRNA translation 10. Most miRNA are first transcribed by RNA polymerase II as pre‐miRNA, which are characterized by hairpin structures and are subsequently processed in the nucleus into long precursor miRNA (pre‐miRNA). The pre‐miRNA are exported to the cytoplasm by exportin‐5 and are further cleaved into mature miRNA 11.

miRNA, which are frequently aberrantly expressed in different types of cancer, have been reported to play a critical role in tumorigenesis, including processes such as proliferation, cell cycle, apoptosis, differentiation, and migration. Recently, emerging evidence has shown that a large group of miRNA were reported to function as key natural regulators in cancer cell metabolism 11. For example, miR‐424 regulates metabolic reprogramming in cancer‐associated fibroblasts by decreasing the expression of IDH3a 12. miR‐181a mediates a metabolic shift in colon cancer cells via the PTEN/AKT pathway 13. miR‐33a and miR‐33b play a critical function in regulating cholesterol homeostasis and fatty acid degradation 14.

Recently, there has been an increasing research interest on the role of miR‐132 in tumorigenesis and cancer treatment. Studies have identified that higher miR‐132 expression could be used as a biomarker of poor prognosis in patients diagnosed with glioma 15. Another group showed that miR‐132 may participate in the tumor progression of osteosarcoma, and loss of miR‐132 expression may be a predictor of unfavorable outcomes in patients with osteosarcoma 16. A report by Zhang B *et al*. found that miR‐132 represses nonsmall cell lung cancer growth by inducing apoptosis, a process that is independent of acetylcholinesterase 17.

However, the direct function of miR‐132 in prostate cancer cells has not been reported. In our study, we report that miR‐132 is significantly reduced in prostate cancer cells. The loss of function of miR‐132 enhances cell proliferation via enhanced glycolysis by directly binding to the 3′‐UTR of Glut1.

## Materials and methods

### Cell culture

PC‐3, DU‐145, and HEK293T cells were purchased from the American Type Culture Collection (ATCC) and cultured in Dulbecco's modified Eagle's medium (DMEM) with 10% heat‐inactivated FBS (Invitrogen, Carlsbad, CA, USA), 100 units·mL^−1^ penicillin/streptomycin and 4.5 g·L^−1^ glucose in a tissue incubator maintained at 37 °C.

### Transfections

All transient transfections were carried out using Lipofectamine 2000 reagent as described previously (Invitrogen) according to the manufacturer's protocol. Transient transfection of microRNA‐132 mimics, microRNA‐132 inhibitor, scramble, and Glut1 siRNA (5′‐UAUUAAAUACAGACACUAA‐3′) at a final concentration of 20 nm was accomplished with lipofectamine 2000 according to the manufacturer's protocol.

### Luciferase reporter assay

The method for the luciferase reporter assay was described previously. Briefly, HEK293T cells were seeded into 96‐well plates and were transfected with 20 nm mimics along with reporter vectors at 100 ng·well^−1^. Luciferase activity was measured after 48 h of transfection using the dual‐luciferase reporter assay system according to the manufacturer's instructions. Luciferase activity was normalized to Renilla luciferase expression. The assay was performed in duplicate, and at least three independent experiments were performed.

### Western blots

Cells were homogenized and lysed in RIPA buffer supplemented with a protease inhibitor cocktail. Protein levels were quantified using the bicinchoninic acid assay kit, and proteins were then separated using an 8% SDS/PAGE gel. Proteins were then transferred to a nitrocellulose membrane, which was blocked with 5% nonfat milk and subsequently exposed to the primary antibody. The indicated proteins were detected with an enhanced chemiluminescence kit.

### Cell viability assays

Cells were seeded at a density of 5000 cells/96‐well in DMEM medium and transfected with miR‐132 mimics or miR‐132 inhibitor. After 48 h, the cells were treated with AlamarBlue Cell Viability Reagent (ThermoFisher Scientific, Grand Island, NY, USA) for 4 h at 37 °C. Cell viability was measured at 560 nm excitation/590 nm emission filter set.

### Colony formation assays

Cells transfected with miR‐132 mimics, miR‐132 inhibitor or scramble controls were examined for colony‐forming ability. Five hundred cells were seeded in six‐well plates and were allowed to grow for up to 7 days without being disturbed. After fixation by 70% ethanol, cells were stained with 0.5% crystal violet, and the cell colonies were subsequently counted.

### Measurement of glucose uptake and extracellular lactate secretion

The miR‐132 mimics or the miR‐132 inhibitor were transiently transfected into prostate cancer cells. The cell medium was harvested after transfection for 48 h. Glucose uptake was measured using a colorimetric assay according to the manufacturer's instructions (Glucose Uptake Colorimetric Assay Kit; BioVision, Milpitas, CA, USA). Lactate production was measured using the lactate assay kit (Sigma‐Aldrich, Saint Louis, MO, USA).

### Analysis of miR‐132 expression in GSE36802 dataset

The NCBI Gene Expression Omnibus database (http://www.ncbi.nlm.nih.gov/geo/) was used to analyze miR‐132 expression in prostate cancer compared with normal tissues 18. Each dataset was log2 transformed, median normalized. The miR‐132 expression level of individual transcripts were subsequently examined across all the sample datasets.

### Statistical analysis

All values were expressed as the mean ± SD. Statistical analysis was performed using prism 5.0 software (GraphPad, San Diego, CA, USA) using a two‐tailed Student's *t*‐test. *P* < 0.05 was considered statistically significant for all analyses.

## Results

### miR‐132 levels are decreased in prostate cancer cells

**Figure 1:**
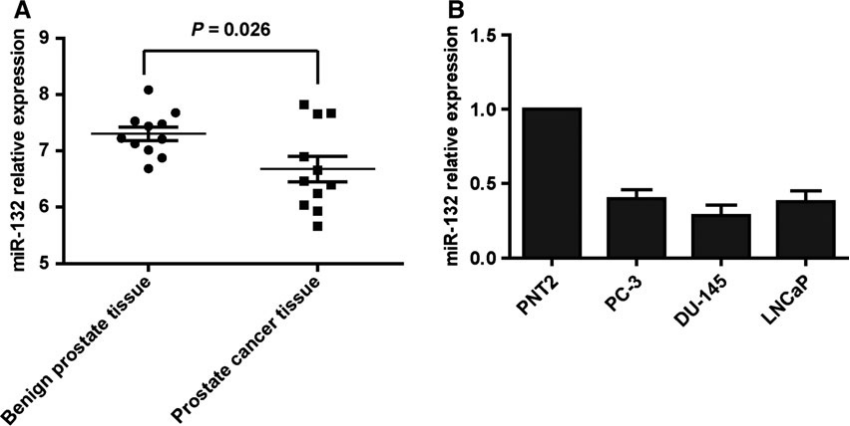
miR‐132 is decreased in prostate cancer. (A) The microRNA expression assay reveals that miR‐132 is significantly decreased in prostate cancer tissue compared with normal tissue. (B) Real‐time PCR analysis shows that miR‐132 expression is significantly decreased in prostate cancer cell lines compared with the normal cell line (*N* = 3).

To explore the expression of miR‐132 in prostate cancer cells, we downloaded a published dataset (Gene expression omnibus accession GSE36802) 18 and analyzed the miR‐132 expression in prostate cancer cells. The data indicated that miR‐132 was markedly lower in prostate cancer tissue compared with normal tissue, with a *P* value of 0.026 (Fig. 1A). Next, we confirmed the expression of miR‐132 in prostate cancer cell lines. qRT‐PCR results showed that miR‐132 expression is significantly lower in prostate cancer cell lines compared with the normal prostate cell line, PNT2 (Fig. 1B).

### miR‐132 suppresses proliferation of prostate cancer cells

**Figure 2:**
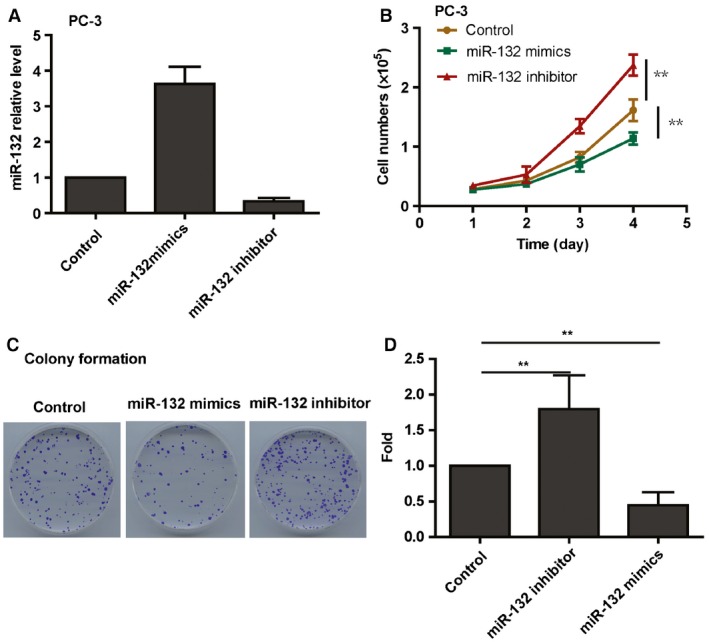
miR‐132 inhibits the proliferation of prostate cancer cells. (A) Real‐time PCR analysis of miR‐132 levels in prostate cancer cells transfected with the miR‐132 mimics or the miR‐132 inhibitor (*N* = 3). (B) The alarma blue assay results show that the growth rate of miR‐132‐overexpressing prostate cancer cells is decreased compared with control cells. Conversely, knockdown of miR‐132 in prostate cancer cells enhances cell growth (*N* = 3). (C) and (D) The colony formation assays show that knockdown of miR‐132 enhances prostate cancer cells colony formation, while the overexpression of miR‐132 decreases colony formation in SAS cells (*N* = 3).

To examine the biological function of miR‐132 on the proliferation of prostate cancer cells, we transfected the miR‐132 mimic or the miR‐132 inhibitor into prostate cancer cells at a total of 20 nm. The qRT‐PCR analysis showed that miR‐132 expression was significantly increased in the miR‐132 mimic‐transfected groups and reduced in the miR‐132 inhibitor‐transfected groups (Fig. 2A). The cell proliferation assay by accounting cell numbers demonstrated that cell growth was decreased in miR‐132 overexpression prostate cancer cells compared with the control cells (Fig. 2B). To further explore whether the inhibition of miR‐132 in prostate cancer cells increased cell growth, knockdown of miR‐132 in prostate cancer cells transfected with the miR‐132 inhibitor enhanced cell growth as expected (Fig. 2B). The colony formation assay also showed that the gain of function of miR‐132 enhances prostate cancer colony formation, while loss of function of miR‐132 inhibits prostate cancer colony formation (Fig. 2C,D). In conclusion, these data suggest that downregulation of miR‐132 enhanced cell growth in prostate cancer cell.

### Reduction in miR‐132 expression induces a metabolic switch in prostate cancer cells

**Figure 3:**
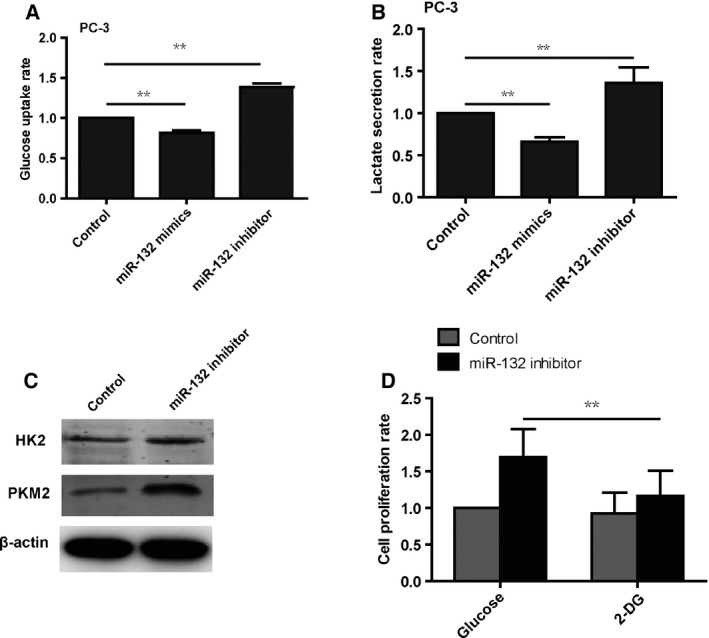
miR‐132 induces a metabolic shift in prostate cancer cells. (A) Glucose uptake assays show that miR‐132 decreases the rate of glucose uptake (*N* = 3). (B) Lactate production assays show that miR‐132 blocks lactate secretion (*N* = 3). (C) Western blot results show that knockdown of miR‐132 in prostate cancer cells with the inhibitor increases the expression of HK2 and PKM2 (*N* = 3). (D) The alarma blue assays show that inhibition of glycolysis with 2‐DG blocks the enhanced proliferation rate induced by knockdown of miR‐132 (*N* = 3).

We further explored whether reduced expression of miR‐132 was capable of inducing a metabolic switch in prostate cancer cells. Knockdown of miR‐132 expression in prostate cancer cells increased glucose uptake rate and lactate secretion (Fig. 3A,B). In contrast, overexpression of miR‐132 in prostate cancer cells transfected with miR‐132 mimics decreased the rate of glucose uptake and lactate secretion (Fig. 3A,B). Western blot analysis showed that, compared with control cells, the HK2 and PKM2 proteins, which are two key proteins involved in glycolysis, were increased in cells treated with the miR‐132 inhibitor (Fig. 3C).

To examine whether a miR‐132‐induced metabolic shift is required for cell proliferation, we treated prostate cancer cells transfected with the miR‐132 inhibitor with 2‐deoxyglucose (2‐DG), a glucose analog that suppresses glycolysis by binding to hexokinase, and subsequently performed a cell proliferation assay with alarmablue assay. The data demonstrated that the repression of glycolysis induced by the miR‐132 inhibitor is sufficient to inhibit cell growth (Fig. 3D). Taken together, these data show that miR‐132‐induced glycolysis is required for increased cell growth.

### miR‐132 represses Glut1 expression by directly targeting its 3′UTR

**Figure 4:**
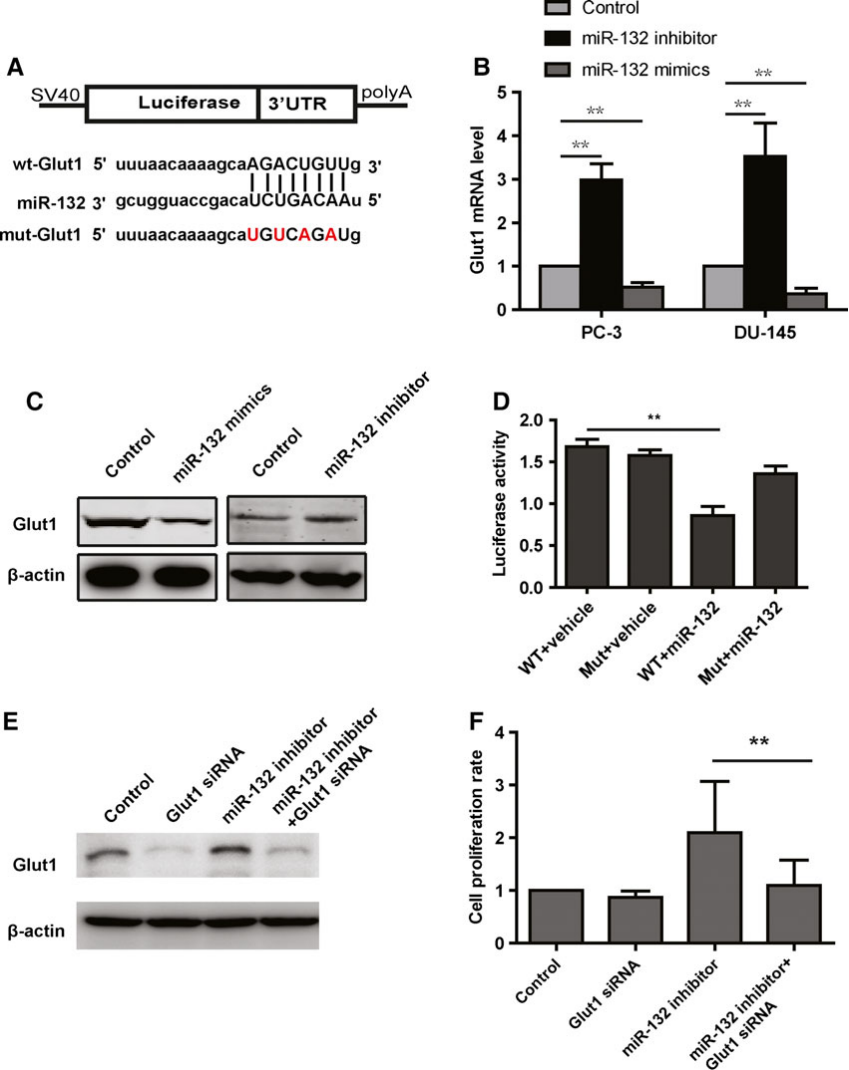
miR‐132 suppresses the expression of Glut1. (A) Sequences of miR‐132 and the potential binding sites at the 3′UTR of Glut1. (B) Western blot analysis shows that overexpression of miR‐132 suppresses the expression of Glut1, while knockdown of miR‐132 increases the expression of Glut1 (*N* = 3). (C) Real‐time PCR analysis shows that knockdown of miR‐132 significantly increases Glut1 mRNA levels (*N* = 3). (D) Dual‐luciferase reporter assay shows that miR‐132 suppresses the luciferase activity in the 3′UTR of the wild‐type group but not in the 3′UTR of the mutant group (*N* = 3). (E) siRNA‐Glut1 obviously decreased the Glut1 protein level in cells transfected with miR‐132 inhibitor. (F) Knockdown of Glut1 suppressed the increased proliferation induced by miR‐132 inhibitor.

We used two different mRNA target‐predicting algorithms (miRanda; TargetScan, www.targetscan.org) to predict the potential targets of miR‐132. Candidate genes involved in glycolysis were of interest. The result showed that an area within the 3′UTR of Glut1 might be a potential target of miR‐132 (Fig. 4A). The data from the western blot analysis confirmed that overexpression of miR‐132 suppressed Glut1 at the protein level (Fig. 4C), while knockdown of miR‐132 increased the expression of Glut1 (Fig. 4C). To explore whether miR‐132 regulated Glut1 at the mRNA level, we analyzed the mRNA levels of Glut1 using real‐time PCR. The results showed that knockdown of miR‐132 significantly increased the Glut1 mRNA levels (Fig. 4B). Next, to test whether Glut1 is a direct downstream target of miR‐132, we performed a luciferase reporter assay. The results indicated that miR‐132 significantly repressed the luciferase activity in the wild‐type group, when the four nucleotides in red were mutated into their complimentary nucleotides (Fig. 4A), miR‐132 could no longer affect the luciferase activity (Fig. 4D). To detect whether Glut1 is required for miR‐132 in regulating cell proliferation, we knockdown the expression of Glut1 in PC3 cells transfected with miR‐132 inhibitor and then analyzed the cell proliferation. The results showed that siRNA obviously decreases Glut1 protein level in PC3 cells treated with miR‐132 inhibitor (Fig. 4E). As expected, the alarma blue assay showed that the increased proliferation rate induced by miR‐132 is suppressed by Glut1 knockdown (Fig. 4F). Taken together, the data indicated that miR‐132 inhibits the expression of Glut1 by directly binding to the 3′UTR.

## Discussion

miRNA have gained increasing attention due to their critical roles in many biological processes, including cell cycle, proliferation, differentiation, cell motility and apoptosis, and in the metabolic switch 19. Several miRNA and their targets have been shown to be abnormally expressed in prostate cancer, leading to the development, invasion, and metastasis of this disease 20. The altered expression of miRNA is useful as biomarkers for the diagnosis, prognosis, and classification of prostate cancer 21, 22. Thus, understanding the characteristic miRNA abnormalities could contribute to the development of novel therapeutic strategies in prostate cancer.

Recently, the aberrant expression of miR‐132 was associated with different types of human cancers. Significant downregulation of miR‐132 has been shown in pituitary tumors, breast cancer, and primary osteosarcoma 16, 23, 24; however, the function of miR‐132 in prostate cancer cell tumorigenesis remains unclear.

In this study, we are the first to report that miR‐132 expression was reduced in prostate cancer tissues compared with adjacent normal tissues. Previous studies have shown that miR‐132 inhibits cell proliferation by acting as a tumor suppressor in osteosarcoma and breast cancer. Consistent with the previous data, our data from the alarma blue assay indicated that miR‐132 reduced cell growth in prostate cancer.

The molecular mechanism underlying altered metabolism is complicated and not fully understood. In our study, we showed that miR‐132 is involved in regulating the aerobic metabolism in prostate cancer cells. Inhibition of miR‐132 enhances lactate secretion and glucose uptake, while overexpression of miR‐132 reduces lactate secretion and glucose uptake. miR‐132‐induced metabolism is required for prostate cancer cell growth. In addition, we showed that Glut1 is a direct downstream target of miR‐132. Downregulation of miR‐132 in prostate cancer cells needs to be extensively investigated for its role in cancer cell metabolism.

In conclusion, our study reveals new insight that reduction in miR‐132 in prostate cancer cells enhances aerobic glycolysis by regulating Glut1 expression, thus promoting cell proliferation. These data identify miR‐132 as a molecule involved in the regulation of the Warburg effect in prostate cancer cells and as a potential therapeutic target for prostate cancer.

## Author contributions

WQ, SD performed most of the experiments; SW, XZ, GL performed some of the experiments. Gang Cao wrote the manuscript. All the authors have read this manuscript.
